# Supplementary material for: Identification of TAPBPL as a novel negative regulator of T‐cell function
Source: EMBO Mol Med. 2021 May 3;13(5):e13404. doi: 10.15252/emmm.202013404 (PMC8103088; doi:10.15252/emmm.202013404)
Supplement: Supplementary file 1 — Appendix [file EMMM-13-e13404-s004.pdf]

APPENDIX

Appendix Figure S1. Characterization of hTAPBPL-Ig protein and anti-hTAPBPL mAb.

Appendix Figure S2. mTAPBPL-Ig protein inhibits mouse T cell activation and proliferation *in vitro*.

Appendix Figure S3. The effects of hTAPBPL-Ig protein on cytokine production from T cells *in vitro*.

Appendix Figure S4. hTAPBPL-Ig prevents EAE development in mice.

Appendix Table 1: Exact P values

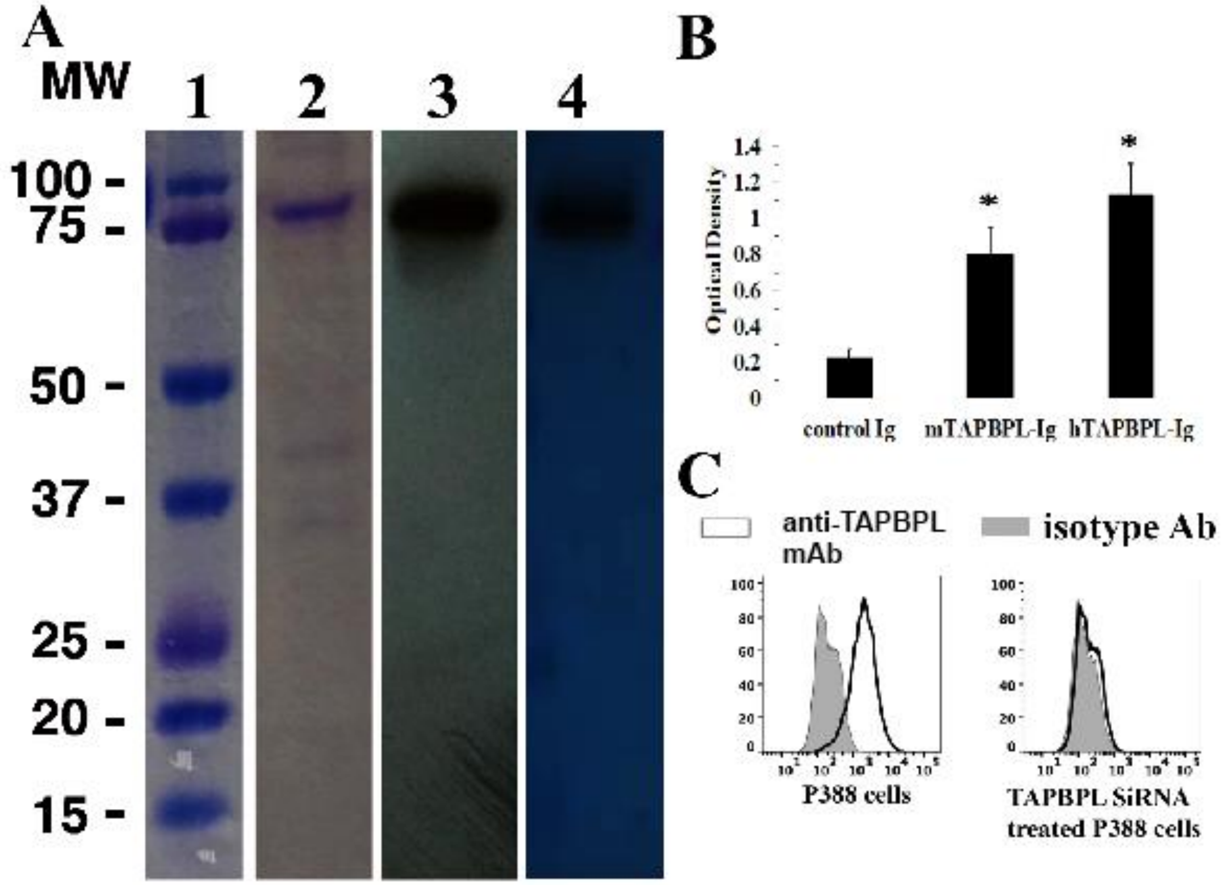

Appendix Figure S1. Characterization of hTAPBPL-Ig protein and anti-hTAPBPL mAb.
A. SDS-PAGE and Western blot for purified hTAPBPL-Ig protein; Lane 1: Molecular weight (MWS1 markers; 2: Coomassie blue-stained SDS-PAGE; 3: Western blot with goat anti-mouse IgG2a antibody. 4: Western blot with anti-hTAPBPL mAb (clone 54).
B. The anti-TAPBPL mAb (clone 54) reacted with hTAPBPL-Ig and mTAPBPL-Ig, but not control Ig protein as detected by ELISA. Statistical significance for optical density between hTAPBPL-Ig or mTAPBPL-Ig and control Ig was determined using one-way ANOVA with Dunnett test (n=3, **P* < 0.05). The data are expressed as mean + SD.
C. The anti-hTAPBPL mAb stained parent P388 leukemia cells, but not mTAPBPL siRNA treated P388 cells.

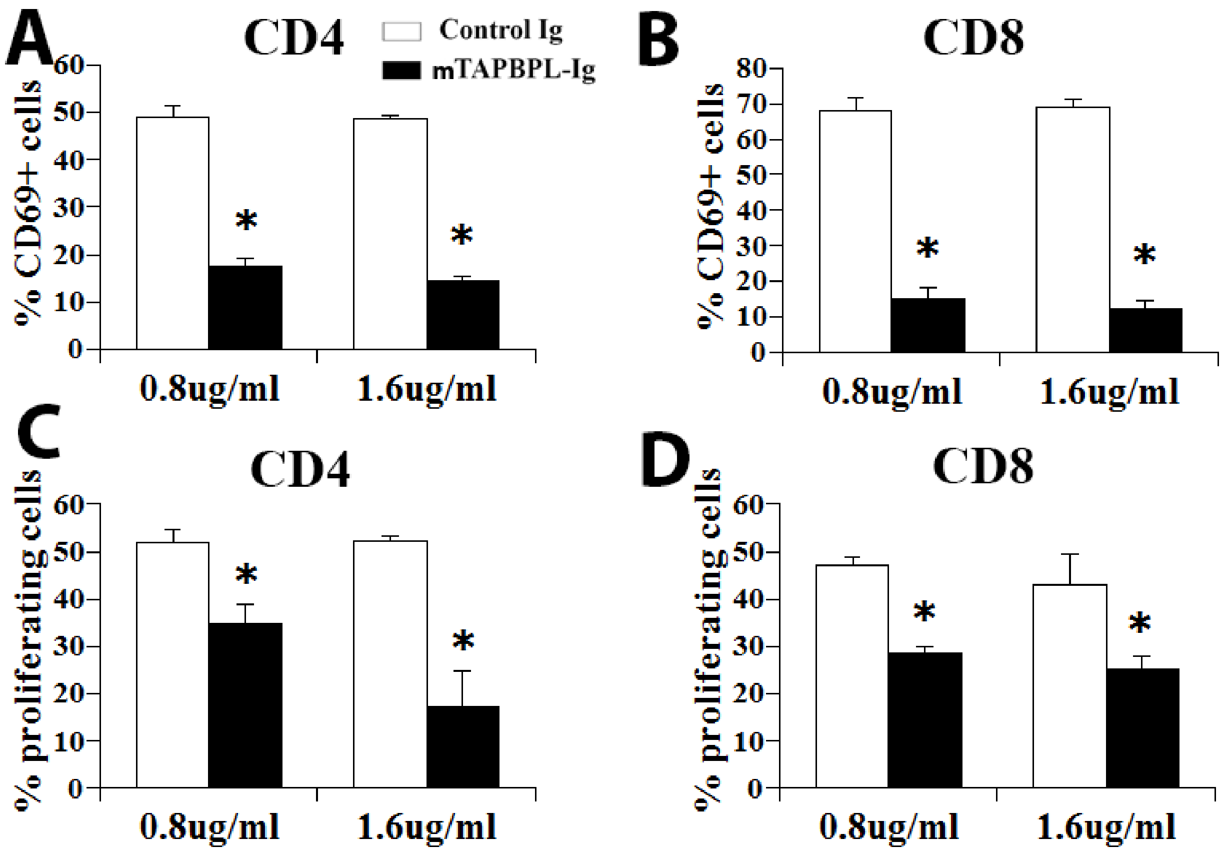

Appendix Figure S2. mTAPBPL-Ig protein inhibits mouse T cell activation and proliferation *in vitro*.

A. B. mTAPBPL-Ig protein inhibits mouse T cell activation. Splenic cells from C57BL/6 mice were cultured with anti-CD3 antibody (1 µg/ml) in the presence of control Ig or mTAPBPL-Ig (0.8 and 1.6 µg/ml) for 1 day. The cells were analyzed for the percentage of $CD69^+$ cells in CD4 and CD8 T cells. Statistical significance between mTAPBPL-Ig- and control Ig-treated cells was determined using two-tailed *t*-test (n=3, $*P < 0.05$). The data are expressed as mean + SD.

C. D. mTAPBPL-Ig protein inhibits mouse T cell proliferation. Splenic cells from C57BL/6 mice were labelled with CFSE and cultured with anti-CD3 antibody (1 µg/ml) in the presence of control Ig or mTAPBPL-Ig (0.8 and 1.6 µg/ml) for 3 day. The cells were analyzed for CFSE levels by $CD4^+$ and $CD8^+$ T cells. Statistical significance between mTAPBPL-Ig- and control Ig-treated cells was determined using two-tailed *t*-test (n=3, $*P < 0.05$). The data are expressed as mean + SD.

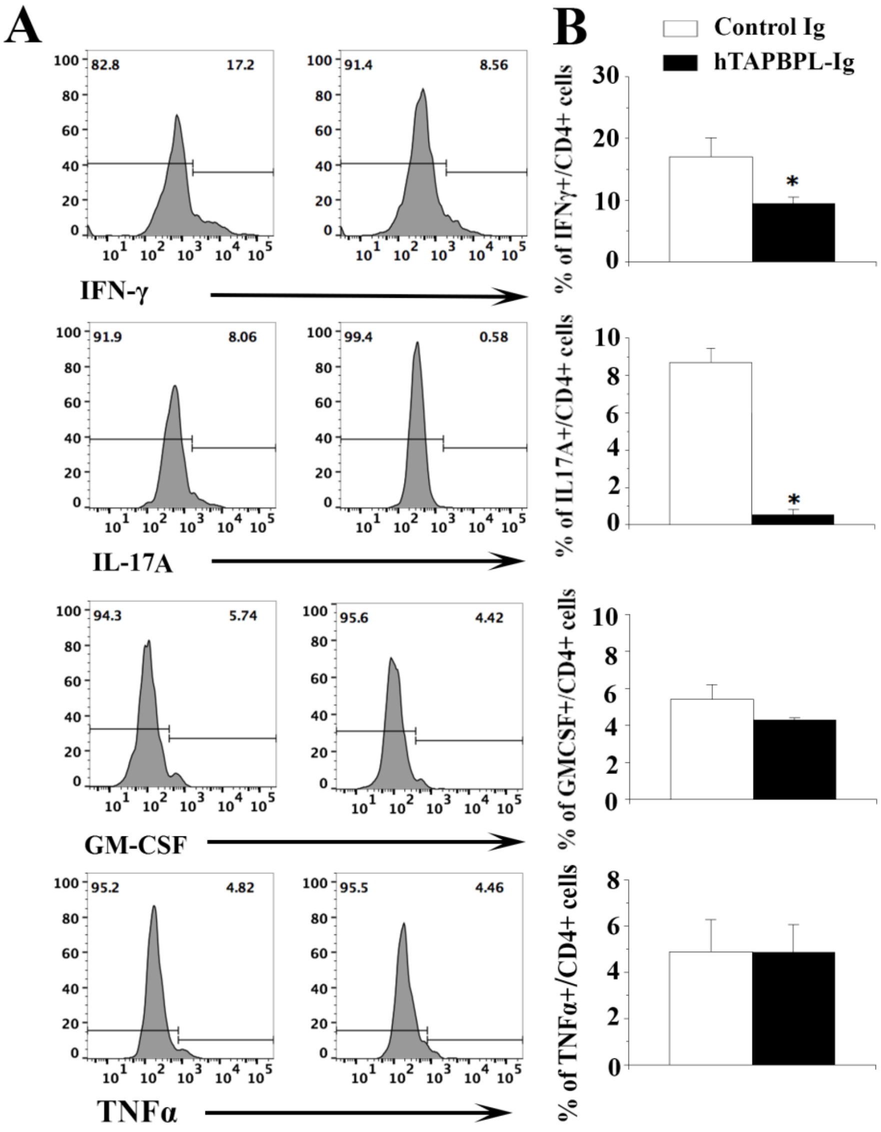

Appendix Figure S3. The effects of hTAPBPL-Ig protein on cytokine production from T cells *in vitro*. Purified murine $CD4^+$ T cells were cultured with plate-bound anti-CD3 antibody (1 µg /ml) in the presence of hTAPBPL-Ig (10 µg/ml) or equimolar amount of control Ig. The cells were stimulated with PMA and ionomycin 4 hours before harvesting and then stained with antibodies against IFNγ, IL-17A, GM-CSF, and TNFα. The percentages of the cytokine-positive cells in $CD4^+$ T cells were determined by flow cytometry. (A) Representative flow cytometric profile, (B) statistical analyses of the percentages of cytokine producing $CD4^+$ T cells. Statistical significance between hTAPBPL-Ig- and control Ig-treated cells was determined using two-tailed *t*-test (n=3, $^*P < 0.05$). The data are expressed as mean $\pm$ SD.

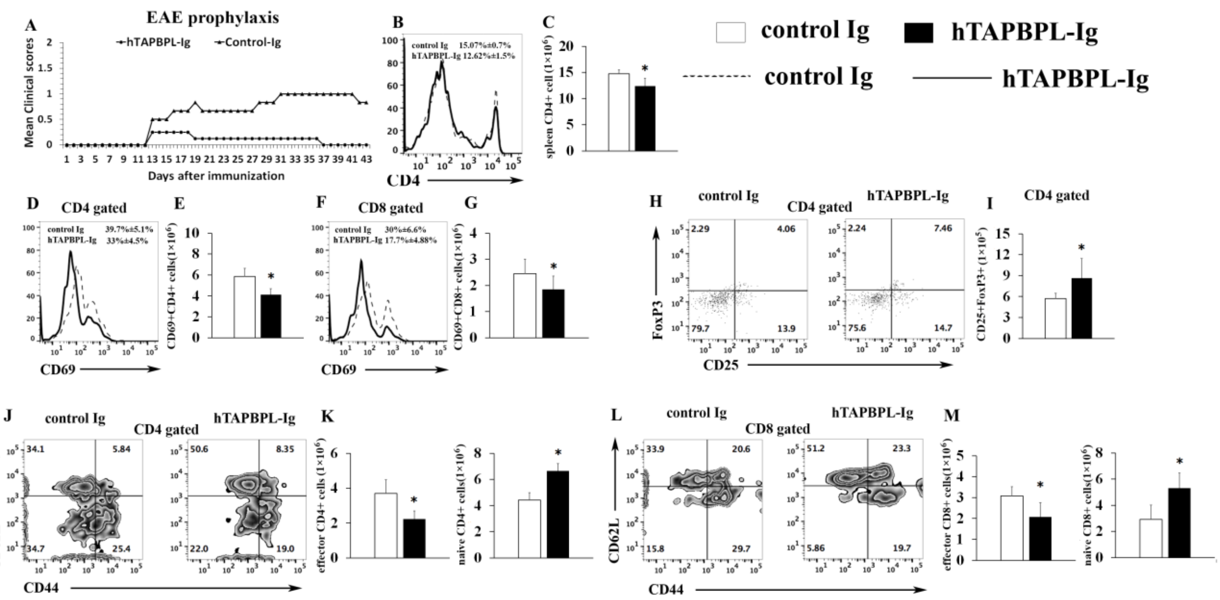

Appendix Figure S4. hTAPBPL-Ig prevents EAE development in mice. C57BL/6 mice were immunized with 200 µg MOG35-55 emulsified in CFA and 500 ng of purified Bordetella pertussis toxin. Mice were injected i.p. with 25 µg hTAPBPL-Ig or control Ig protein 3 times per week from day 0. EAE development was monitored. (A) Mean clinical scores. On day 43 post immunization, the spleens were harvested and analyzed for the (B, D, F, H, J, L) percentage and (C, E, G, I, K, M) number of (B, C) CD4$^+$ T cells, (D, E) CD4$^+$CD69$^+$ T cells, (F, G) CD8$^+$CD69$^+$ T cells, (H and I) CD4$^+$CD25$^+$FoxP3$^+$ Tregs, as well as CD44^{hi}CD62L^{lo} effector memory and CD44^{lo}CD62L^{hi} naïve (J, K) CD4$^+$ T cells and (L, M) CD8$^+$ T cells. The data are expressed as mean $\pm$ SD (n= 10/group). Significance was calculated by two-tailed Student's *t*-test. * P<0.05 compared with control Ig.

Appendix Table 1: Exact P values

Figure	Panel	Sub-panel	Compared groups	P-value
Figure 2	B	CD4	anti-TAPBPL Ab for resting cells vs isotype Ab for resting cells	0.0001
			anti-TAPBPL Ab for activated cells vs isotype Ab for activated cells	<0.0001
			anti-TAPBPL Ab for activated cells vs anti-TAPBPL Ab for resting cells	NS
		CD8	anti-TAPBPL Ab for resting cells vs isotype Ab for resting cells	0.0394
			anti-TAPBPL Ab for activated cells vs isotype Ab for activated cells	<0.0001
			anti-TAPBPL Ab for activated cells vs anti-TAPBPL Ab for resting cells	0.0001
		CD11b	anti-TAPBPL Ab for resting cells vs isotype Ab for resting cells	0.0395
			anti-TAPBPL Ab for activated cells vs isotype Ab for activated cells	<0.0001
			anti-TAPBPL Ab for activated cells vs anti-TAPBPL Ab for resting cells	0.0007
		F4/80	anti-TAPBPL Ab for resting cells vs isotype Ab for resting cells	<0.0001
			anti-TAPBPL Ab for activated cells vs isotype Ab for activated cells	<0.0001
			anti-TAPBPL Ab for activated cells vs anti-TAPBPL Ab for resting cells	NS
		CD11c	anti-TAPBPL Ab for resting cells vs isotype Ab for resting cells	0.0003
			anti-TAPBPL Ab for activated cells vs isotype Ab for activated cells	<0.0001
			anti-TAPBPL Ab for activated cells vs anti-TAPBPL Ab for resting cells	0.0122
		CD19	anti-TAPBPL Ab for resting cells vs isotype Ab for resting cells	<0.0001
			anti-TAPBPL Ab for activated cells vs isotype Ab for activated cells	<0.0001
			anti-TAPBPL Ab for activated cells vs anti-TAPBPL Ab for resting cells	NS
		B220	anti-TAPBPL Ab for resting cells vs isotype Ab for resting cells	<0.0001
			anti-TAPBPL Ab for activated cells vs isotype Ab for activated cells	<0.0001
			anti-TAPBPL Ab for activated cells vs anti-TAPBPL Ab for resting cells	NS
	D		Breast cancer vs normal tissue	0.0302
			Colon cancer vs normal tissue	0.0210
			liver cancer vs normal tissue	0.0239
			lung cancer vs normal tissue	0.0114
			prostate cancer vs normal tissue	0.0479
	F	Neruo-2a	anti-TAPBPL vs isotype Ab	0.0195
		Lewis lung	anti-TAPBPL vs isotype Ab	0.0114
		P388	anti-TAPBPL vs isotype Ab	0.0102
		CT26	anti-TAPBPL vs isotype Ab	0.0057
		B16	anti-TAPBPL vs isotype Ab	0.0367
Figure 3	B	CD4	TAPBPL Ig for resting cells vs Control Ig for resting cells	0.0180

			TAPBPL Ig for activated cells vs TAPBPL Ig for resting cells	0.0202
			TAPBPL Ig for activated cells vs Control Ig for activated cells	0.0002
		CD8	TAPBPL Ig for resting cells vs Control Ig for resting cells	0.0101
			TAPBPL Ig for activated cells vs TAPBPL Ig for resting cells	0.0003
			TAPBPL Ig for activated cells vs Control Ig for activated cells	<0.0001
		CD11b	TAPBPL Ig for resting cells vs Control Ig for resting cells	<0.0001
			TAPBPL Ig for activated cells vs TAPBPL Ig for resting cells	0.0065
			TAPBPL Ig for activated cells vs Control Ig for activated cells	<0.0001
		F4/80	TAPBPL Ig for resting cells vs Control Ig for resting cells	0.0455
			TAPBPL Ig for activated cells vs TAPBPL Ig for resting cells	0.0040
			TAPBPL Ig for activated cells vs Control Ig for activated cells	0.0003
		CD11c	TAPBPL Ig for resting cells vs Control Ig for resting cells	0.0229
			TAPBPL Ig for activated cells vs TAPBPL Ig for resting cells	0.0050
			TAPBPL Ig for activated cells vs Control Ig for activated cells	0.0001
		CD19	TAPBPL Ig for resting cells vs Control Ig for resting cells	<0.0001
			TAPBPL Ig for activated cells vs TAPBPL Ig for resting cells	0.0002
			TAPBPL Ig for activated cells vs Control Ig for activated cells	<0.0001
		B220	TAPBPL Ig for resting cells vs Control Ig for resting cells	<0.0001
			TAPBPL Ig for activated cells vs TAPBPL Ig for resting cells	0.0002
			TAPBPL Ig for activated cells vs Control Ig for activated cells	0.0003
Figure 4	B	CD4 10ug/ml	TAPBPL Ig vs Control Ig	0.0011
		CD4 15ug/ml	TAPBPL Ig vs Control Ig	0.0001
		CD8 10ug/ml	TAPBPL Ig vs Control Ig	0.0008
		CD8 15ug/ml	TAPBPL Ig vs Control Ig	0.0001
	D	CD4 10ug/ml	TAPBPL Ig vs Control Ig	0.0001
		CD4 15ug/ml	TAPBPL Ig vs Control Ig	<0.0001
		CD8 10ug/ml	TAPBPL Ig vs Control Ig	0.0012
		CD8 15ug/ml	TAPBPL Ig vs Control Ig	<0.0001
	F	CD4 10ug/ml	TAPBPL Ig vs Control Ig	0.0062
		CD4 15ug/ml	TAPBPL Ig vs Control Ig	0.0087
		CD8 10ug/ml	TAPBPL Ig vs Control Ig	0.0268
		CD8 15ug/ml	TAPBPL Ig vs Control Ig	0.0458
	H	CD4 10ug/ml	TAPBPL Ig vs Control Ig	0.0011
		CD4 15ug/ml	TAPBPL Ig vs Control Ig	0.0004
		CD8 10ug/ml	TAPBPL Ig vs Control Ig	0.0014
		CD8 15ug/ml	TAPBPL Ig vs Control Ig	0.0070
	J	CD4 10ug/ml	TAPBPL Ig vs Control Ig	<0.0001
		CD4 15ug/ml	TAPBPL Ig vs Control Ig	0.0001

		CD8 10ug/ml	TAPBPL Ig vs Control Ig	<0.0001
		CD8 15ug/ml	TAPBPL Ig vs Control Ig	<0.0001
	L	CD4 10ug/ml	TAPBPL Ig vs Control Ig	0.0002
		CD4 15ug/ml	TAPBPL Ig vs Control Ig	<0.0001
		CD8 10ug/ml	TAPBPL Ig vs Control Ig	0.0001
		CD8 15ug/ml	TAPBPL Ig vs Control Ig	<0.0001
Figure 5	A	10ug/ml	TAPBPL Ig vs Control Ig	0.0011
		15ug/ml	TAPBPL Ig vs Control Ig	<0.0001
	B	10ug/ml	TAPBPL Ig vs Control Ig	0.0010
		15ug/ml	TAPBPL Ig vs Control Ig	0.0001
	D	5ug/ml	TAPBPL Ig vs Control Ig	0.0008
		10ug/ml	TAPBPL Ig vs Control Ig	0.0002
		15ug/ml	TAPBPL Ig vs Control Ig	<0.0001
	E	5ug/ml	TAPBPL Ig vs Control Ig	NS
		10ug/ml	TAPBPL Ig vs Control Ig	0.0098
		15ug/ml	TAPBPL Ig vs Control Ig	0.0019
	F	1.5ug/ml	TAPBPL Ig vs Control Ig	0.0025
		3ug/ml	TAPBPL Ig vs Control Ig	0.0303
Figure 6	A	Day0	TAPBPL Ig vs Control Ig	NS
		Day1	TAPBPL Ig vs Control Ig	NS
		Day2	TAPBPL Ig vs Control Ig	NS
		Day3	TAPBPL Ig vs Control Ig	NS
		Day4	TAPBPL Ig vs Control Ig	NS
		Day5	TAPBPL Ig vs Control Ig	NS
		Day6	TAPBPL Ig vs Control Ig	NS
		Day7	TAPBPL Ig vs Control Ig	NS
		Day8	TAPBPL Ig vs Control Ig	NS
		Day9	TAPBPL Ig vs Control Ig	NS
		Day10	TAPBPL Ig vs Control Ig	NS
		Day11	TAPBPL Ig vs Control Ig	NS
		Day12	TAPBPL Ig vs Control Ig	0.0016
		Day13	TAPBPL Ig vs Control Ig	0.0016
		Day14	TAPBPL Ig vs Control Ig	0.0018
		Day15	TAPBPL Ig vs Control Ig	0.0442
		Day16	TAPBPL Ig vs Control Ig	0.0176
		Day17	TAPBPL Ig vs Control Ig	0.0235
		Day18	TAPBPL Ig vs Control Ig	0.0077
		Day19	TAPBPL Ig vs Control Ig	0.0151
		Day20	TAPBPL Ig vs Control Ig	0.0151
		Day21	TAPBPL Ig vs Control Ig	NS
		Day22	TAPBPL Ig vs Control Ig	NS
		Day23	TAPBPL Ig vs Control Ig	NS
		Day24	TAPBPL Ig vs Control Ig	0.0120
		Day25	TAPBPL Ig vs Control Ig	0.0063
		Day26	TAPBPL Ig vs Control Ig	0.0203
		Day27	TAPBPL Ig vs Control Ig	0.0258
		Day28	TAPBPL Ig vs Control Ig	0.0258
		Day29	TAPBPL Ig vs Control Ig	0.0258
	C		TAPBPL Ig vs Control Ig	<0.0001

	E		TAPBPL Ig vs Control Ig	0.0117
	G		TAPBPL Ig vs Control Ig	0.0434
	I		TAPBPL Ig vs Control Ig	0.0408
	K	effector cells	TAPBPL Ig vs Control Ig	<0.0001
		naïve cells	TAPBPL Ig vs Control Ig	0.0001
	M	effector cells	TAPBPL Ig vs Control Ig	0.0315
		naïve cells	TAPBPL Ig vs Control Ig	0.0430
	O		TAPBPL Ig vs Control Ig	0.0228
	Q		TAPBPL Ig vs Control Ig	0.0151
	R	Day 0	TAPBPL Ig vs Control Ig	NS
			TAPBPL Ig + anti-CD25 Ab vs TAPBPL Ig + Isotype Ab	NS
		Day 1	TAPBPL Ig vs Control Ig	NS
			TAPBPL Ig + anti-CD25 Ab vs TAPBPL Ig + Isotype Ab	NS
		Day 2	TAPBPL Ig vs Control Ig	NS
			TAPBPL Ig + anti-CD25 Ab vs TAPBPL Ig + Isotype Ab	NS
		Day 3	TAPBPL Ig vs Control Ig	0.0369
			TAPBPL Ig + anti-CD25 Ab vs TAPBPL Ig + Isotype Ab	NS
		Day 4	TAPBPL Ig vs Control Ig	0.0369
			TAPBPL Ig + anti-CD25 Ab vs TAPBPL Ig + Isotype Ab	NS
		Day 5	TAPBPL Ig vs Control Ig	0.0196
			TAPBPL Ig + anti-CD25 Ab vs TAPBPL Ig + Isotype Ab	NS
		Day 6	TAPBPL Ig vs Control Ig	NS
			TAPBPL Ig + anti-CD25 Ab vs TAPBPL Ig + Isotype Ab	NS
		Day 7	TAPBPL Ig vs Control Ig	NS
			TAPBPL Ig + anti-CD25 Ab vs TAPBPL Ig + Isotype Ab	NS
		Day 8	TAPBPL Ig vs Control Ig	NS
			TAPBPL Ig + anti-CD25 Ab vs TAPBPL Ig + Isotype Ab	NS
		Day 9	TAPBPL Ig vs Control Ig	NS
			TAPBPL Ig + anti-CD25 Ab vs TAPBPL Ig + Isotype Ab	NS
		Day 10	TAPBPL Ig vs Control Ig	0.0391
			TAPBPL Ig + anti-CD25 Ab vs TAPBPL Ig + Isotype Ab	NS
		Day 11	TAPBPL Ig vs Control Ig	0.0029
			TAPBPL Ig + anti-CD25 Ab vs TAPBPL Ig + Isotype Ab	NS
		Day 12	TAPBPL Ig vs Control Ig	<0.0001
			TAPBPL Ig + anti-CD25 Ab vs TAPBPL Ig + Isotype Ab	0.0103
		Day 13	TAPBPL Ig vs Control Ig	<0.0001
			TAPBPL Ig + anti-CD25 Ab vs TAPBPL Ig + Isotype Ab	0.0051
		Day 14	TAPBPL Ig vs Control Ig	<0.0001
			TAPBPL Ig + anti-CD25 Ab vs TAPBPL Ig + Isotype Ab	0.0103
		Day 15	TAPBPL Ig vs Control Ig	<0.0001
			TAPBPL Ig + anti-CD25 Ab vs TAPBPL Ig + Isotype Ab	0.0103
	S	5ug/ml	TAPBPL Ig vs Control Ig	0.0010
		10ug/ml	TAPBPL Ig vs Control Ig	<0.0001
		15ug/ml	TAPBPL Ig vs Control Ig	<0.0001
Figure 7	A		anti-TAPBPL vs isotype Ab, 12.5ug	0.0156
			anti-TAPBPL vs isotype Ab, 6.25ug	0.0014
	C	CD69+/CD4+	anti-TAPBPL vs isotype Ab, 12.5ug	0.0002
			anti-TAPBPL vs isotype Ab, 25ug	<0.0001
			anti-TAPBPL vs isotype Ab,50ug	<0.0001

		CD69+/CD8+	anti-TAPBPL vs isotype Ab, 12.5ug	<0.0001
			anti-TAPBPL vs isotype Ab, 25ug	<0.0001
			anti-TAPBPL vs isotype Ab,50ug	<0.0001
	D	25ug Day 0	anti-TAPBPL vs isotype Ab	NS
		25ug Day 1	anti-TAPBPL vs isotype Ab	NS
		25ug Day 2	anti-TAPBPL vs isotype Ab	NS
		25ug Day 3	anti-TAPBPL vs isotype Ab	NS
		25ug Day 4	anti-TAPBPL vs isotype Ab	NS
		25ug Day 5	anti-TAPBPL vs isotype Ab	NS
		25ug Day 6	anti-TAPBPL vs isotype Ab	NS
		25ug Day 7	anti-TAPBPL vs isotype Ab	NS
		25ug Day 8	anti-TAPBPL vs isotype Ab	NS
		25ug Day 9	anti-TAPBPL vs isotype Ab	NS
		25ug Day 10	anti-TAPBPL vs isotype Ab	NS
		25ug Day 11	anti-TAPBPL vs isotype Ab	NS
		25ug Day 12	anti-TAPBPL vs isotype Ab	NS
		25ug Day 13	anti-TAPBPL vs isotype Ab	NS
		25ug Day 14	anti-TAPBPL vs isotype Ab	NS
		25ug Day 15	anti-TAPBPL vs isotype Ab	0.0391
		50ug Day 0	anti-TAPBPL vs isotype Ab	NS
		50ug Day 1	anti-TAPBPL vs isotype Ab	NS
		50ug Day 2	anti-TAPBPL vs isotype Ab	NS
		50ug Day 3	anti-TAPBPL vs isotype Ab	NS
		50ug Day 4	anti-TAPBPL vs isotype Ab	NS
		50ug Day 5	anti-TAPBPL vs isotype Ab	NS
		50ug Day 6	anti-TAPBPL vs isotype Ab	NS
		50ug Day 7	anti-TAPBPL vs isotype Ab	NS
		50ug Day 8	anti-TAPBPL vs isotype Ab	NS
		50ug Day 9	anti-TAPBPL vs isotype Ab	NS
		50ug Day 10	anti-TAPBPL vs isotype Ab	NS
		50ug Day 11	anti-TAPBPL vs isotype Ab	NS
		50ug Day 12	anti-TAPBPL vs isotype Ab	NS
		50ug Day 13	anti-TAPBPL vs isotype Ab	NS
		50ug Day 14	anti-TAPBPL vs isotype Ab	NS
		50ug Day 15	anti-TAPBPL vs isotype Ab	NS
		100ug Day 0	anti-TAPBPL vs isotype Ab	NS
		100ug Day 1	anti-TAPBPL vs isotype Ab	NS
		100ug Day 2	anti-TAPBPL vs isotype Ab	NS
		100ug Day 3	anti-TAPBPL vs isotype Ab	NS
		100ug Day 4	anti-TAPBPL vs isotype Ab	NS
		100ug Day 5	anti-TAPBPL vs isotype Ab	NS
		100ug Day 6	anti-TAPBPL vs isotype Ab	NS
		100ug Day 7	anti-TAPBPL vs isotype Ab	NS
		100ug Day 8	anti-TAPBPL vs isotype Ab	NS
		100ug Day 9	anti-TAPBPL vs isotype Ab	NS
		100ug Day 10	anti-TAPBPL vs isotype Ab	NS
		100ug Day 11	anti-TAPBPL vs isotype Ab	0.0335
		100ug Day 12	anti-TAPBPL vs isotype Ab	NS
		100ug Day 13	anti-TAPBPL vs isotype Ab	0.0087

		100ug Day 14	anti-TAPBPL vs isotype Ab	0.0087
		100ug Day 15	anti-TAPBPL vs isotype Ab	0.0051
	F		anti-TAPBPL vs isotype Ab	0.0000
	H		anti-TAPBPL vs isotype Ab	0.0000
	J		anti-TAPBPL vs isotype Ab	0.0139
	M	100ug Day 0	anti-TAPBPL vs isotype Ab	NS
		100ug Day 1	anti-TAPBPL vs isotype Ab	NS
		100ug Day 2	anti-TAPBPL vs isotype Ab	NS
		100ug Day 3	anti-TAPBPL vs isotype Ab	NS
		100ug Day 4	anti-TAPBPL vs isotype Ab	NS
		100ug Day 5	anti-TAPBPL vs isotype Ab	NS
		100ug Day 6	anti-TAPBPL vs isotype Ab	NS
		100ug Day 7	anti-TAPBPL vs isotype Ab	NS
		100ug Day 8	anti-TAPBPL vs isotype Ab	NS
		100ug Day 9	anti-TAPBPL vs isotype Ab	NS
		100ug Day 10	anti-TAPBPL vs isotype Ab	NS
		100ug Day 11	anti-TAPBPL vs isotype Ab	NS
		100ug Day 12	anti-TAPBPL vs isotype Ab	0.0139
		100ug Day 13	anti-TAPBPL vs isotype Ab	0.0001
		100ug Day 14	anti-TAPBPL vs isotype Ab	0.0005
		100ug Day 15	anti-TAPBPL vs isotype Ab	0.0001
EV1	A	Monocytes	anti-TAPBPL vs isotype Ab, resting	0.0052
			anti-TAPBPL vs isotype Ab,2.5ug/ml LPS	0.0035
			anti-TAPBPL vs isotype Ab,5ug/ml LPS	NS
			anti-TAPBPL vs isotype Ab,10ug/ml LPS	0.0449
			anti-TAPBPL vs isotype Ab,50ng/ml IFNγ	<0.0001
			anti-TAPBPL vs isotype Ab,100ug/ml IFNγ	0.0006
		DCs	anti-TAPBPL vs isotype Ab, resting	0.0033
			anti-TAPBPL vs isotype Ab,2.5ug/ml LPS	0.0222
			anti-TAPBPL vs isotype Ab,5ug/ml LPS	<0.0001
			anti-TAPBPL vs isotype Ab,10ug/ml LPS	0.0001
			anti-TAPBPL vs isotype Ab,50ng/ml IFNγ	0.0007
			anti-TAPBPL vs isotype Ab,100ug/ml IFNγ	0.0010
	B	CD4	activated cells vs resting cells	NS
		CD8	activated cells vs resting cells	0.0195
		CD11b	activated cells vs resting cells	0.0137
		F4/80	activated cells vs resting cells	NS
		CD11c	activated cells vs resting cells	0.0057
		CD19	activated cells vs resting cells	NS
	E	CD19	anti-TAPBPL Ab for resting cells vs isotype Ab for resting cells	0.0485
			anti-TAPBPL Ab for activated cells vs isotype Ab for activated cells	<0.0001
			anti-TAPBPL Ab for activated cells vs anti-TAPBPL Ab for resting cells	0.0010
		CD14	anti-TAPBPL Ab for resting cells vs isotype Ab for resting cells	<0.0001
			anti-TAPBPL Ab for activated cells vs isotype Ab for activated cells	<0.0001

			anti-TAPBPL Ab for activated cells vs anti-TAPBPL Ab for resting cells	<0.0001
		DCs	anti-TAPBPL Ab for resting cells vs isotype Ab for resting cells	NS
			anti-TAPBPL Ab for activated cells vs isotype Ab for activated cells	<0.0001
			anti-TAPBPL Ab for activated cells vs anti-TAPBPL Ab for resting cells	<0.0001
	H	Neuro-2a	IFNγ vs W/O IFNγ	0.0004
		Lewis lung	IFNγ vs W/O IFNγ	NS
		P388	IFNγ vs W/O IFNγ	NS
		CT26	IFNγ vs W/O IFNγ	NS
		B16	IFNγ vs W/O IFNγ	<0.0001
EV2	CD4	resting	TAPBPL-Ig vs control Ig	0.0310
		anti-CD3 0.5ug/ml + anti-CD28 0.25ug/ml	TAPBPL-Ig vs control Ig	0.0144
		anti-CD3 1ug/ml + anti-CD28 0.5ug/ml	TAPBPL-Ig vs control Ig	0.0001
		anti-CD3 2ug/ml + anti-CD28 1ug/ml	TAPBPL-Ig vs control Ig	<0.0001
	CD8	resting	TAPBPL-Ig vs control Ig	0.0057
		anti-CD3 0.5ug/ml + anti-CD28 0.25ug/ml	TAPBPL-Ig vs control Ig	0.0011
		anti-CD3 1ug/ml + anti-CD28 0.5ug/ml	TAPBPL-Ig vs control Ig	0.0162
		anti-CD3 2ug/ml + anti-CD28 1ug/ml	TAPBPL-Ig vs control Ig	0.0185
	CD11b	resting	TAPBPL-Ig vs control Ig	0.0022
		2.5ug/ml LPS	TAPBPL-Ig vs control Ig	0.0003
		5ug/ml LPS	TAPBPL-Ig vs control Ig	<0.0001
		10ug/ml LPS	TAPBPL-Ig vs control Ig	0.0083
	F/80	resting	TAPBPL-Ig vs control Ig	0.0006
		2.5ug/ml LPS	TAPBPL-Ig vs control Ig	0.0005
		5ug/ml LPS	TAPBPL-Ig vs control Ig	0.0087
		10ug/ml LPS	TAPBPL-Ig vs control Ig	0.0076
	CD11c	resting	TAPBPL-Ig vs control Ig	0.0135
		2.5ug/ml LPS	TAPBPL-Ig vs control Ig	0.0007
		5ug/ml LPS	TAPBPL-Ig vs control Ig	0.0091
		10ug/ml LPS	TAPBPL-Ig vs control Ig	0.0027
EV3	B	p-AKT 10min	hTAPBPL Ig vs Control Ig	NS
			PD-1 Ig vs Control Ig	NS
		p-AKT 30min	hTAPBPL Ig vs Control Ig	0.0070
			PD-1 Ig vs Control Ig	0.0119
		pAKT 60min	hTAPBPL Ig vs Control Ig	0.0263
			PD-1 Ig vs Control Ig	0.0420
		p-p38 10min	hTAPBPL Ig vs Control Ig	0.0489

			PD-1 Ig vs Control Ig	NS
		p-p38 30min	hTAPBPL Ig vs Control Ig	0.0007
			PD-1 Ig vs Control Ig	NS
		p-p38 60min	hTAPBPL Ig vs Control Ig	0.0496
			PD-1 Ig vs Control Ig	NS
		p-JNK 10min	hTAPBPL Ig vs Control Ig	NS
			PD-1 Ig vs Control Ig	NS
		p-JNK 30min	hTAPBPL Ig vs Control Ig	NS
			PD-1 Ig vs Control Ig	NS
		p-JNK 60min	hTAPBPL Ig vs Control Ig	0.0246
			PD-1 Ig vs Control Ig	NS
EV4	A	IFN-γ	hTAPBPL Ig vs Control Ig	0.0001
		IL-17A	hTAPBPL Ig vs Control Ig	0.0023
	B	Microglias cells	hTAPBPL Ig vs Control Ig	NS
		Macrophages	hTAPBPL Ig vs Control Ig	NS
		Neutrophils	hTAPBPL Ig vs Control Ig	NS
	E	5ug/ml	hTAPBPL Ig vs Control Ig	0.0014
		10ug/ml	hTAPBPL Ig vs Control Ig	0.0026
	F	IFN-γ	hTAPBPL Ig vs Control Ig	<0.0001
		IL-17A	hTAPBPL Ig vs Control Ig	0.0001
	G	IFN-γ	hTAPBPL Ig vs Control Ig	<0.0001
		IL-17A	hTAPBPL Ig vs Control Ig	<0.0001
		IL-2	hTAPBPL Ig vs Control Ig	NS
		GM-CSF	hTAPBPL Ig vs Control Ig	NS
EV5	A	CD4,6.25ug/ml	anti-TAPBPL vs isotype Ab	NS
		CD8,6.25ug/ml	anti-TAPBPL vs isotype Ab	NS
		CD4,12.5ug/ml	anti-TAPBPL vs isotype Ab	NS
		CD8,12.5ug/ml	anti-TAPBPL vs isotype Ab	NS
	B	CD4,6.25ug/ml	anti-TAPBPL vs isotype Ab	NS
		CD8,6.25ug/ml	anti-TAPBPL vs isotype Ab	NS
		CD4,12.5ug/ml	anti-TAPBPL vs isotype Ab	NS
		CD8,12.5ug/ml	anti-TAPBPL vs isotype Ab	NS
	C	25ug, Day 0	anti-TAPBPL vs isotype Ab	NS
		25ug, Day 1	anti-TAPBPL vs isotype Ab	NS
		25ug, Day 2	anti-TAPBPL vs isotype Ab	NS
		25ug, Day 3	anti-TAPBPL vs isotype Ab	NS
		25ug, Day 4	anti-TAPBPL vs isotype Ab	NS
		25ug, Day 5	anti-TAPBPL vs isotype Ab	NS
		25ug, Day 6	anti-TAPBPL vs isotype Ab	NS
		25ug, Day 7	anti-TAPBPL vs isotype Ab	NS
		25ug, Day 8	anti-TAPBPL vs isotype Ab	NS
		25ug, Day 9	anti-TAPBPL vs isotype Ab	NS
		25ug, Day 10	anti-TAPBPL vs isotype Ab	NS
		25ug, Day 11	anti-TAPBPL vs isotype Ab	NS
		25ug, Day 12	anti-TAPBPL vs isotype Ab	NS
		25ug, Day 13	anti-TAPBPL vs isotype Ab	NS
		25ug, Day 14	anti-TAPBPL vs isotype Ab	0.0300
		25ug, Day 15	anti-TAPBPL vs isotype Ab	0.0498
		50ug, Day 0	anti-TAPBPL vs isotype Ab	NS

		50ug, Day 1	anti-TAPBPL vs isotype Ab	NS
		50ug, Day 2	anti-TAPBPL vs isotype Ab	NS
		50ug, Day 3	anti-TAPBPL vs isotype Ab	NS
		50ug, Day 4	anti-TAPBPL vs isotype Ab	NS
		50ug, Day 5	anti-TAPBPL vs isotype Ab	NS
		50ug, Day 6	anti-TAPBPL vs isotype Ab	NS
		50ug, Day 7	anti-TAPBPL vs isotype Ab	NS
		50ug, Day 8	anti-TAPBPL vs isotype Ab	NS
		50ug, Day 9	anti-TAPBPL vs isotype Ab	NS
		50ug, Day 10	anti-TAPBPL vs isotype Ab	NS
		50ug, Day 11	anti-TAPBPL vs isotype Ab	NS
		50ug, Day 12	anti-TAPBPL vs isotype Ab	NS
		50ug, Day 13	anti-TAPBPL vs isotype Ab	NS
		50ug, Day 14	anti-TAPBPL vs isotype Ab	NS
		50ug, Day 15	anti-TAPBPL vs isotype Ab	NS
		100ug, Day 0	anti-TAPBPL vs isotype Ab	NS
		100ug, Day 1	anti-TAPBPL vs isotype Ab	NS
		100ug, Day 2	anti-TAPBPL vs isotype Ab	NS
		100ug, Day 3	anti-TAPBPL vs isotype Ab	NS
		100ug, Day 4	anti-TAPBPL vs isotype Ab	NS
		100ug, Day 5	anti-TAPBPL vs isotype Ab	NS
		100ug, Day 6	anti-TAPBPL vs isotype Ab	NS
		100ug, Day 7	anti-TAPBPL vs isotype Ab	NS
		100ug, Day 8	anti-TAPBPL vs isotype Ab	NS
		100ug, Day 9	anti-TAPBPL vs isotype Ab	NS
		100ug, Day 10	anti-TAPBPL vs isotype Ab	NS
		100ug, Day 11	anti-TAPBPL vs isotype Ab	NS
		100ug, Day 12	anti-TAPBPL vs isotype Ab	NS
		100ug, Day 13	anti-TAPBPL vs isotype Ab	NS
		100ug, Day 14	anti-TAPBPL vs isotype Ab	0.0498
		100ug, Day 15	anti-TAPBPL vs isotype Ab	0.0170
	E	CD4	anti-TAPBPL vs isotype Ab	0.0002
		CD8	anti-TAPBPL vs isotype Ab	0.0124
	F	CD4	anti-TAPBPL vs isotype Ab	<0.0001
	G	CD8	anti-TAPBPL vs isotype Ab	<0.0001
	H	T reg	anti-TAPBPL vs isotype Ab	0.0006
	I	Day0	anti-TAPBPL Ab + anti-CD8 Ab vs anti-TAPBPL Ab + anti-CD8 isotype Ab	NS
		Day1	anti-TAPBPL Ab + anti-CD8 Ab vs anti-TAPBPL Ab + anti-CD8 isotype Ab	NS
		Day2	anti-TAPBPL Ab + anti-CD8 Ab vs anti-TAPBPL Ab + anti-CD8 isotype Ab	NS
		Day3	anti-TAPBPL Ab + anti-CD8 Ab vs anti-TAPBPL Ab + anti-CD8 isotype Ab	NS
		Day4	anti-TAPBPL Ab + anti-CD8 Ab vs anti-TAPBPL Ab + anti-CD8 isotype Ab	NS
		Day5	anti-TAPBPL Ab + anti-CD8 Ab vs anti-TAPBPL Ab + anti-CD8 isotype Ab	NS
		Day6	anti-TAPBPL Ab + anti-CD8 Ab vs anti-TAPBPL Ab + anti-CD8 isotype Ab	NS

		Day7	anti-TAPBPL Ab + anti-CD8 Ab vs anti-TAPBPL Ab + anti-CD8 isotype Ab	NS
		Day8	anti-TAPBPL Ab + anti-CD8 Ab vs anti-TAPBPL Ab + anti-CD8 isotype Ab	NS
		Day9	anti-TAPBPL Ab + anti-CD8 Ab vs anti-TAPBPL Ab + anti-CD8 isotype Ab	NS
		Day10	anti-TAPBPL Ab + anti-CD8 Ab vs anti-TAPBPL Ab + anti-CD8 isotype Ab	NS
		Day11	anti-TAPBPL Ab + anti-CD8 Ab vs anti-TAPBPL Ab + anti-CD8 isotype Ab	NS
		Day12	anti-TAPBPL Ab + anti-CD8 Ab vs anti-TAPBPL Ab + anti-CD8 isotype Ab	NS
		Day13	anti-TAPBPL Ab + anti-CD8 Ab vs anti-TAPBPL Ab + anti-CD8 isotype Ab	NS
		Day14	anti-TAPBPL Ab + anti-CD8 Ab vs anti-TAPBPL Ab + anti-CD8 isotype Ab	NS
		Day15	anti-TAPBPL Ab + anti-CD8 Ab vs anti-TAPBPL Ab + anti-CD8 isotype Ab	<0.0001
	J	Day 0	anti-TAPBPL vs isotype Ab	NS
		Day 1	anti-TAPBPL vs isotype Ab	NS
		Day 2	anti-TAPBPL vs isotype Ab	NS
		Day 3	anti-TAPBPL vs isotype Ab	NS
		Day 4	anti-TAPBPL vs isotype Ab	NS
		Day 5	anti-TAPBPL vs isotype Ab	NS
		Day 6	anti-TAPBPL vs isotype Ab	NS
		Day 7	anti-TAPBPL vs isotype Ab	NS
		Day 8	anti-TAPBPL vs isotype Ab	NS
		Day 9	anti-TAPBPL vs isotype Ab	NS
		Day 10	anti-TAPBPL vs isotype Ab	NS
		Day 11	anti-TAPBPL vs isotype Ab	NS
		Day 12	anti-TAPBPL vs isotype Ab	NS
		Day 13	anti-TAPBPL vs isotype Ab	NS
		Day 14	anti-TAPBPL vs isotype Ab	0.0102
		Day 15	anti-TAPBPL vs isotype Ab	0.0177
Appendix S1	B		mTAPBPL Ig vs Control Ig	<0.0001
			hTAPBPL Ig vs Control Ig	<0.0001
Appendix S2	A	0.8ug/ml	mTAPBPL Ig vs Control Ig	<0.0001
		1.6ug/ml	mTAPBPL Ig vs Control Ig	<0.0001
	B	0.8ug/ml	mTAPBPL Ig vs Control Ig	<0.0001
		1.6ug/ml	mTAPBPL Ig vs Control Ig	<0.0001
	C	0.8ug/ml	mTAPBPL Ig vs Control Ig	0.0223
		1.6ug/ml	mTAPBPL Ig vs Control Ig	0.0003
	D	0.8ug/ml	mTAPBPL Ig vs Control Ig	0.0072
		1.6ug/ml	mTAPBPL Ig vs Control Ig	0.0227
Appendix S3	B	IFNγ	hTAPBPL Ig vs Control Ig	0.0019
		IL-17A	hTAPBPL Ig vs Control Ig	<0.0001
		GMCSF	hTAPBPL Ig vs Control Ig	NS
		TNFα	hTAPBPL Ig vs Control Ig	NS
Appendix S4	C		hTAPBPL Ig vs Control Ig	0.0282
	E		hTAPBPL Ig vs Control Ig	<0.0001
	G		hTAPBPL Ig vs Control Ig	0.0155

	I		hTAPBPL Ig vs Control Ig	0.0412
	K	effector	hTAPBPL Ig vs Control Ig	<0.0001
		Naïve	hTAPBPL Ig vs Control Ig	<0.0001
	M	effector	hTAPBPL Ig vs Control Ig	0.0054
		Naïve	hTAPBPL Ig vs Control Ig	0.0001
