# Supplementary material for: Identification of TAPBPL as a novel negative regulator of T‐cell function
Source: EMBO Mol Med. 2021 May 3;13(5):e13404. doi: 10.15252/emmm.202013404 (PMC8103088; doi:10.15252/emmm.202013404)
Supplement: Supplementary file 2 — Expanded View Figures PDF [file EMMM-13-e13404-s001.pdf]

Expanded View Figures

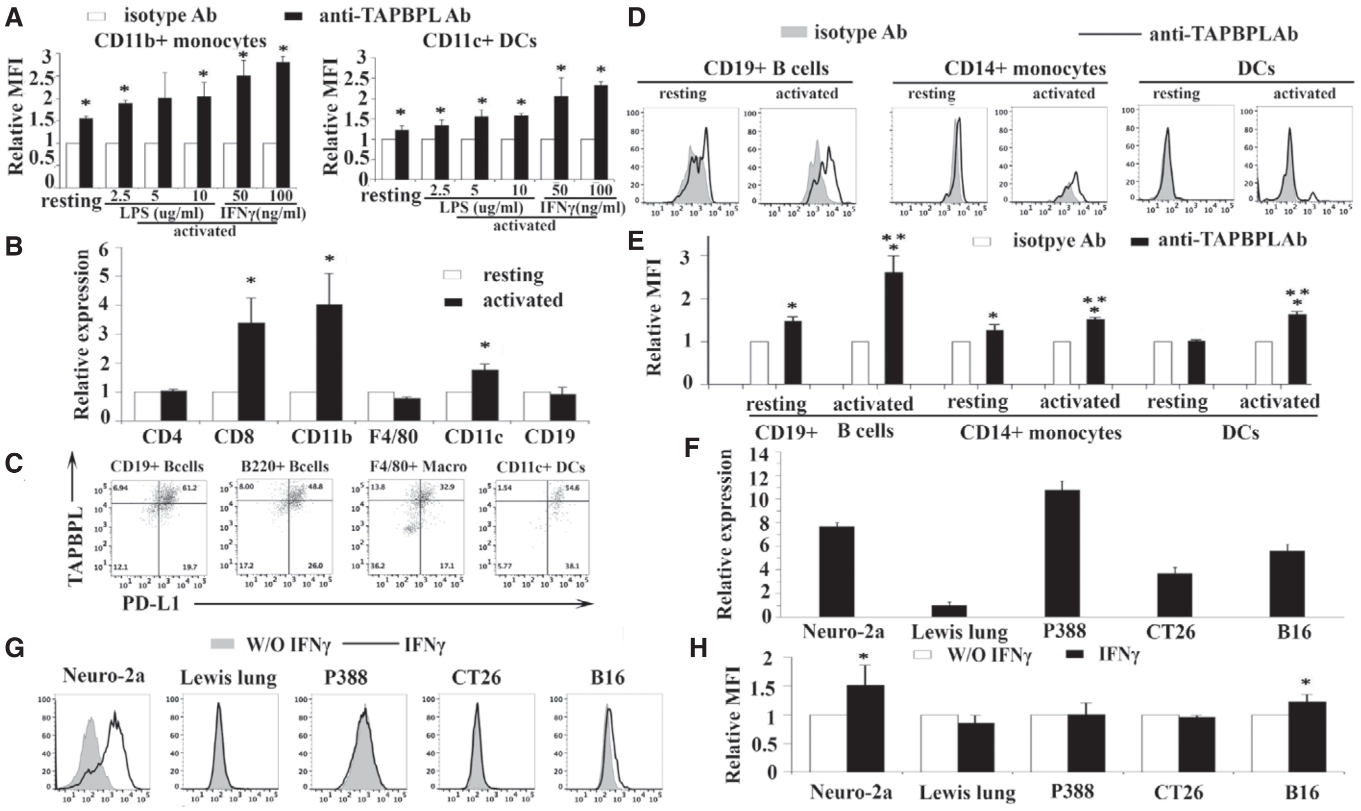

Figure EV1. Analysis of TAPBPL mRNA and protein expression on murine and human immune cells, as well as cancer cells.

A Splenocytes from C57BL/6 mice were incubated with different doses of LPS (2.5, 5, 10 μg/ml) or IFNγ (50, 100 ng/ml) for 3 days. The activated immune cells were stained with anti-TAPBPL or isotype antibody (Ab) and anti-CD11b or CD11c Ab, and analyzed by flow cytometry. Freshly harvested resting immune cells were used as controls. Data show the expression levels of TAPBPL protein on resting and activated CD11b$^+$ monocytes or CD11c$^+$ DCs (n = 3). *P < 0.05 compared with isotype Ab.

B The expression pattern of TAPBPL mRNA in immune cells. CD4$^+$ and CD8$^+$ T cells, CD11b$^+$ monocytes, F4/80$^+$ macrophages, CD11c$^+$ DCs, and CD19$^+$ B cells were magnetically isolated from splenocytes of C57BL/6 mice. To activate T cells, purified CD4$^+$ or CD8$^+$ T cells were incubated with anti-CD3 (1 μg/ml) and anti-CD28 (0.5 μg/ml) antibodies for 3 days. To activate B cells, monocytes, macrophages, or DCs, the purified immune cells were incubated with LPS (10 μg/ml) for 3 days. RNA was isolated from the resting and activated immune cells. The expression levels of TAPBPL mRNA in the cells were determined by qRT–PCR (n = 3). Relative expression levels are shown, and the expression levels in resting cells are defined as 1. *P < 0.05 compared with resting immune cells.

C The co-expression of TAPBPL and PD-L1 on activated APCs. Splenocytes from C57BL/6 mice were incubated with LPS (10 μg/ml) for 3 days. The cells were stained with anti-TAPBPL and anti-PD-L1 Abs, as well as anti-CD19, B220, F4/80, and CD11c Abs. Representative flow cytometric profiles showing the expression of TAPBPL and PD-L1 on APCs.

D, E The expression of TAPBPL protein on resting and activated human CD19$^+$ B cells and CD14$^+$ monocytes, as well as immature and mature DCs in PBMCs, was analyzed by flow cytometry. To activate the immune cells, the cells were stimulated with IFNγ (100 ng/ml) and LPS (1 μg/ml). (D) Representative flow cytometric profiles and (E) statistical analysis (n = 3), *P < 0.05 compared with isotype Ab, **P < 0.05 compared with resting cells.

F The expression pattern of TAPBPL mRNA in cancer cells. RNA was isolated from the indicated cancer cells. The expression levels of TAPBPL mRNA in the cells were determined by qRT–PCR. The expression level in Lewis lung cancer cells was defined as 1. The data are representative of 3 independent experiments.

G, H The expression of TAPBPL on tumor cells following IFNγ stimulation. The indicated tumor cells were incubated with 20 ng/ml IFNγ for 2 days and then analyzed for the expression of TAPBPL by flow cytometry. (G) Representative flow cytometric profiles and (H) statistical analysis (n = 3), *P < 0.05 compared with unstimulated cells.

Data information: Significance in (E) was calculated by two-way ANOVA with Tukey test and others by two-tailed Student's t-test.

 © 2021 The Authors

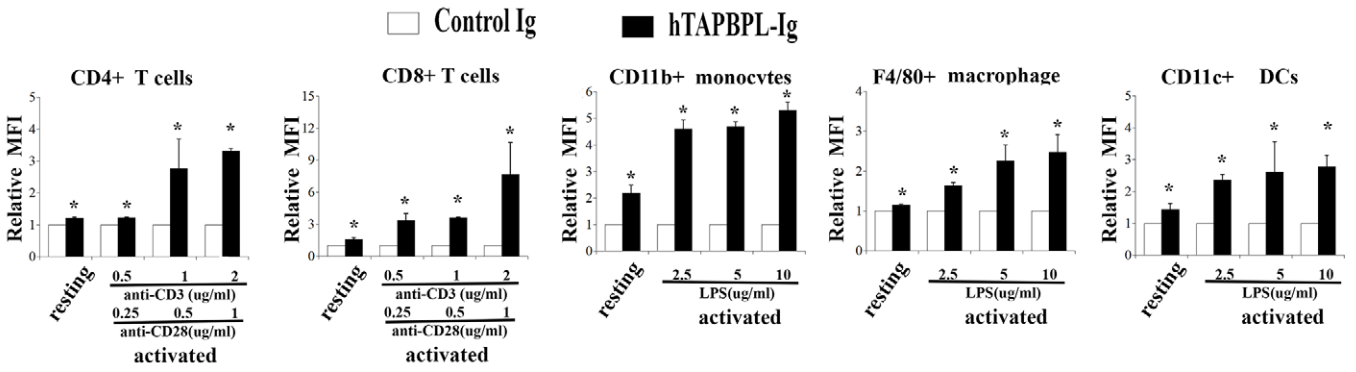

Figure EV2. The expression levels of the putative TAPBPL receptor on activated immune cells with different doses of stimulators.

Splenocytes from C57BL/6 mice were incubated with different doses of anti-CD3 (0.5, 1, 2 μg/ml) and anti-CD28 (0.25, 0.5, 1 μg/ml) antibodies, or LPS (2.5, 5, 10 μg/ml) for 3 days. The activated immune cells were stained with biotinylated TAPBPL-Ig or control Ig, followed by streptavidin-PE, as well as antibodies against immune cell surface markers. Freshly harvested resting immune cells were used as controls. Statistical analysis showing the binding TAPBPL-Ig or control Ig to resting and activated immune cells ($n = 3$). Significance was calculated by two-tailed Student's t-test. $^*P < 0.05$ compared with control Ig.

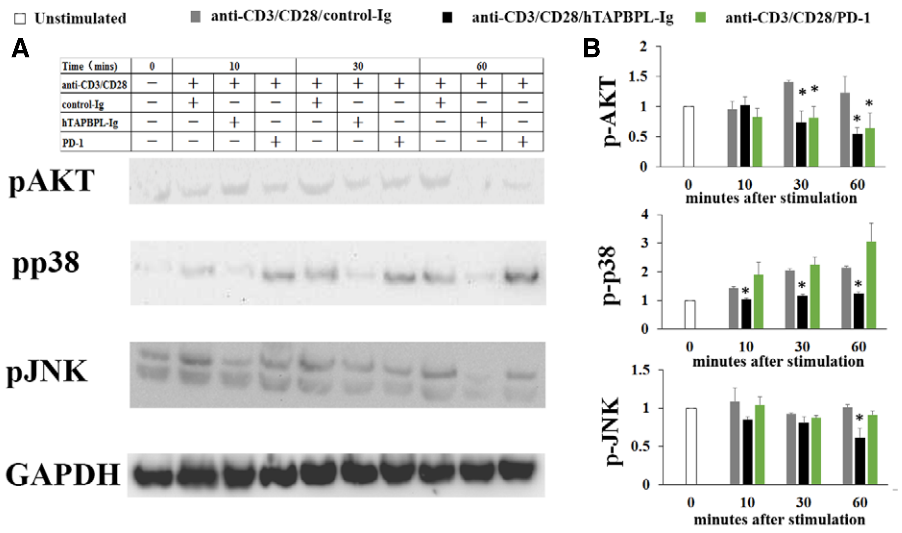

Figure EV3. TAPBPL and PD-L1 affect different signaling molecules in T cells.

CD3+ T cells were purified from C57BL/6 mice and stimulated with anti-CD3 (1 μg/ml) and anti-CD28 (0.5 μg/ml) antibodies in the presence of control Ig, hTAPBPL-Ig, or PD-L1-Ig for indicted times. The expression of the indicated proteins was analyzed by Western blot.

A, B (A) Representative figures and (B) densitometric analysis of changes in the expression levels normalized to that of GAPDH. The data are expressed as the fold change relative to mean values before stimulation ($n = 3$). Significance was calculated by one-way ANOVA with Dunnett test. $^*P < 0.05$ compared with control Ig group.

Source data are available online for this figure.

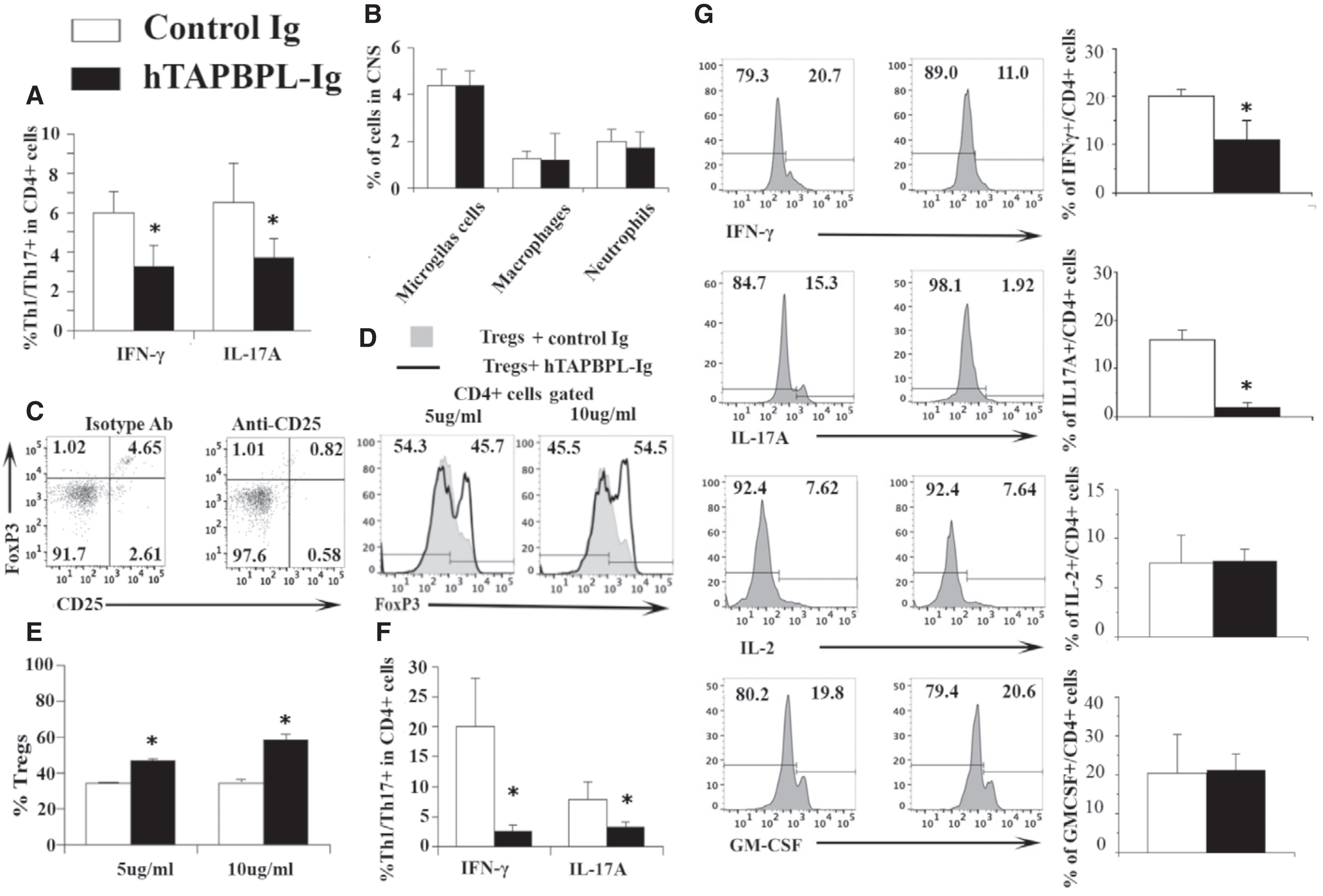

Figure EV4. Further characterization of immune cells in hTAPBPL-Ig-treated EAE mice.

A, B C57BL/6 mice were induced to develop EAE and treated with hTAPBPL-Ig or control Ig protein; the spinal cord was harvested at the end of the studies as in Fig 6. The spinal cord was analyzed for the percentages of (A) IFNγ- or IL-17A-producing cells in $CD4^+$ T cells and (B) microglial cells, macrophages, and neutrophils in the hTAPBPL-Ig and control Ig-treated mice by flow cytometry.

C Depletion of Tregs in EAE mice by anti-CD25 antibody. C57BL/6 mice were induced to develop EAE as in Fig 6. Once EAE symptoms occurred, the mice were injected i.p. with 250 μg anti-CD25 antibody (clone PC61, from BioXCell) or isotype antibody on days 0 and +4. Representative flow cytometric profiles showing the percentage of $CD4^+CD25^+FoxP3^+$ Tregs in the blood of the mice on day 6.

D, E $CD4^+CD25^-$ T cells isolated from C57BL/6 mice and induced to differentiate into Tregs in the presence of hTAPBPL-Ig (5 and 10 μg/ml) or equimolar amounts of control Ig for 5 days. The percentage of $CD4^+CD25^+Foxp3^+$ Tregs was analyzed by flow cytometry. (D) Representative flow cytometric profiles and (E) statistical significance between hTAPBPL-Ig- and control Ig-treated cells.

F C57BL/6 mice were induced to develop EAE and treated with hTAPBPL-Ig or control Ig protein; the spleen was harvested at the end of the studies. The splenocytes were stimulated with MOG *in vitro* as in Fig 6S. The percentage of IFNγ- or IL-17A-producing cells in $CD4^+$ T cells was analyzed by flow cytometry.

G hTAPBPL-Ig treatment reduces Th1/Th17 cytokine production from $CD4^+$ T cells. C57BL/6 mice were induced to develop EAE and treated with hTAPBPL-Ig or control Ig protein; the draining lymph nodes were harvested at the end of the studies as in Fig 6. Single-cell suspension of the lymph nodes was cultured with MOG35-55 for 72 h and analyzed for the percentages of IFNγ-, IL-17A-, IL-2-, and GM-CSF-producing $CD4^+$ T cells. Left: Representative flow cytometric profiles. Right: Statistical analysis of the draining lymph nodes. Statistical significance between hTAPBPL-Ig- and control Ig-treated groups was analyzed using two-tailed *t*-test (A, B, F, G: $n = 6$; E: $n = 3$; $^*P < 0.05$ compared with control Ig).

 © 2021 The Authors

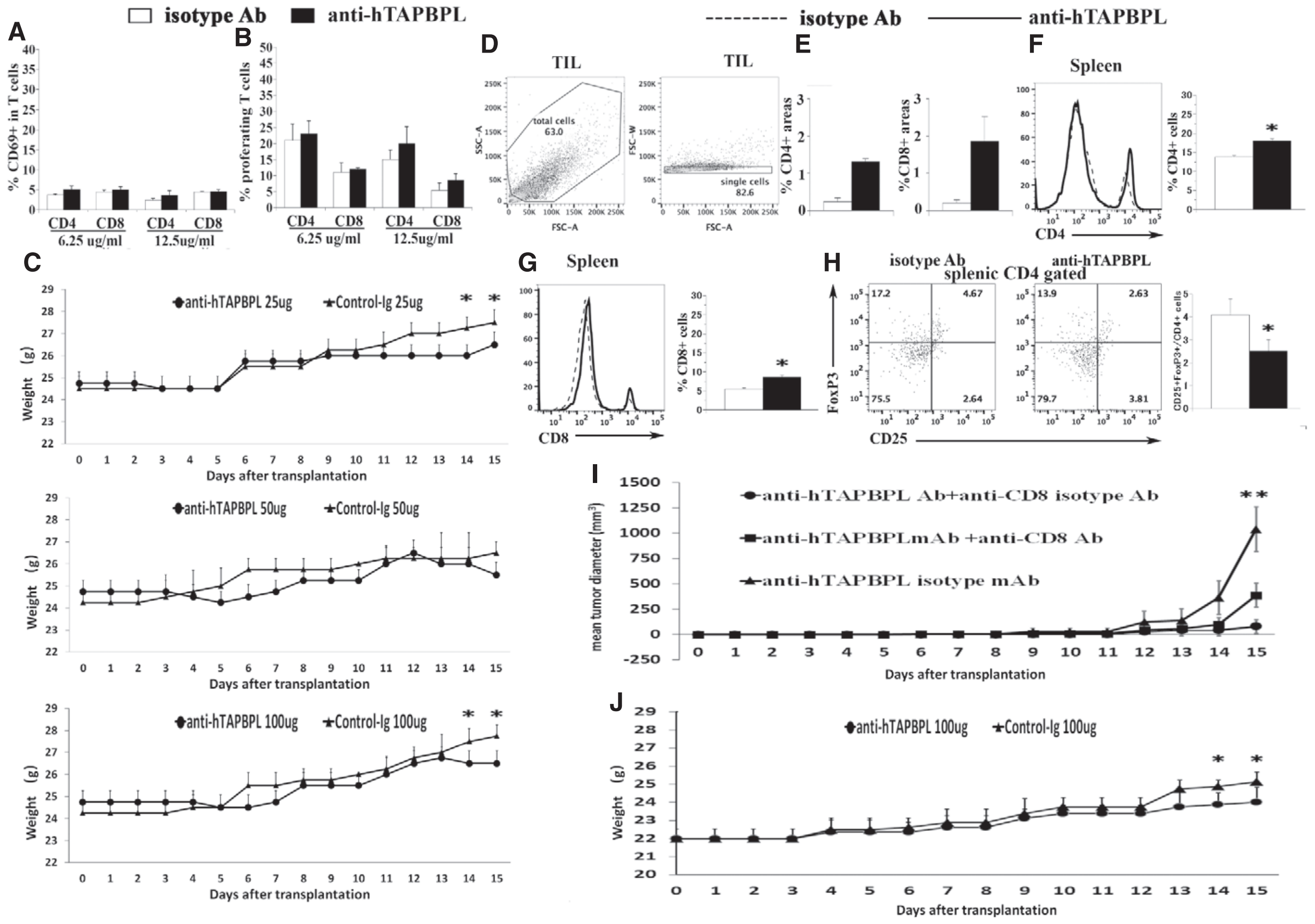

Figure EV5. Anti-hTAPBPL mAb inhibits tumor growth *in vivo*.

A The anti-hTAPBPL mAb alone does not affect CD69 expression on purified T cells *in vitro*. Purified mouse $CD3^+$ T cells were cultured with anti-hTAPBPL mAb (6.25 or 12.5 μg/ml) or isotype Ab (6.25 or 12.5 μg/ml) for 1 day. The percentage of $CD69^+$ in $CD4^+$ or CD8 T^+ cells was analyzed by flow cytometry ($n = 3$).

B The anti-hTAPBPL mAb alone does not affect the proliferation of purified T cells *in vitro*. Purified mouse $CD3^+$ T cells were labeled with CFSE and cultured with anti-hTAPBPL mAb (6.25 or 12.5 μg/ml) or isotype Ab (6.25 or 12.5 μg/ml) for 3 days. The cells were analyzed for CFSE levels by $CD4^+$ and $CD8^+$ T cells ($n = 3$).

C The body weight of the mice in Fig 7D.

D The gating strategy for tumor-infiltrating $CD4^+$ and $CD8^+$ T cells in Fig 7E and G.

E Quantification of $CD4^+$ and $CD8^+$ T-cell areas for Fig 7K and L.

F–H Anti-hTAPBPL mAb-treated tumor-bearing mice have increased T cells and decreased Tregs in the spleen. DBA/2J mice were injected s.c. with P388 murine leukemia cells, followed by injection of the anti-hTAPBPL mAb or isotype Ab (100 μg) 3 times per week as in Fig 7D. At the end of the studies, the spleens were harvested and analyzed for the percentage of (F) $CD4^+$ T cells, (G) $CD8^+$ T cells, and (H) $CD4^+$ $CD25^+$ $FoxP3^+$ Tregs by flow cytometry. The data are expressed as mean + SD and representative of 2 independent experiments with similar results ($n = 6$/group/time).

I Anti-CD8 antibody partly abrogates the antitumor activity of the anti-TAPBPL mAb. DBA/2J mice were injected s.c. with P388 murine leukemia cells, followed by injection of the anti-hTAPBPL mAb or isotype Ab (100 μg) 3 times per week as in Fig 7D. The mice were also injected i.p. with anti-CD8 Ab or isotype Ab (500 μg/injection) on days −3, −1, and + 4 of P388 cell injection ($n = 6$/group/time).

J The body weight of the mice in Fig 7M.

Data information: Significance in (I) was calculated by two-way ANOVA with Tukey test and others by two-tailed Student's *t*-test. *$P < 0.05$ compared with isotype antibody. (I) **$P < 0.05$, anti-TAPBPL Ab + anti-CD8 Ab group was compared with anti-TAPBPL Ab + anti-CD8 isotype Ab group.

© 2021 The Authors
