# Supplementary material for: Identification of TAPBPL as a novel negative regulator of T‐cell function
Source: EMBO Mol Med. 2021 May 3;13(5):e13404. doi: 10.15252/emmm.202013404 (PMC8103088; doi:10.15252/emmm.202013404)
Supplement: Supplementary file 3 — Source Data for Expanded View [file EMMM-13-e13404-s003.zip › emmm202013404-sup-0003-SDataEV/emmm202013404-sup-0003-SDataEV.pptx]

## Slide 1
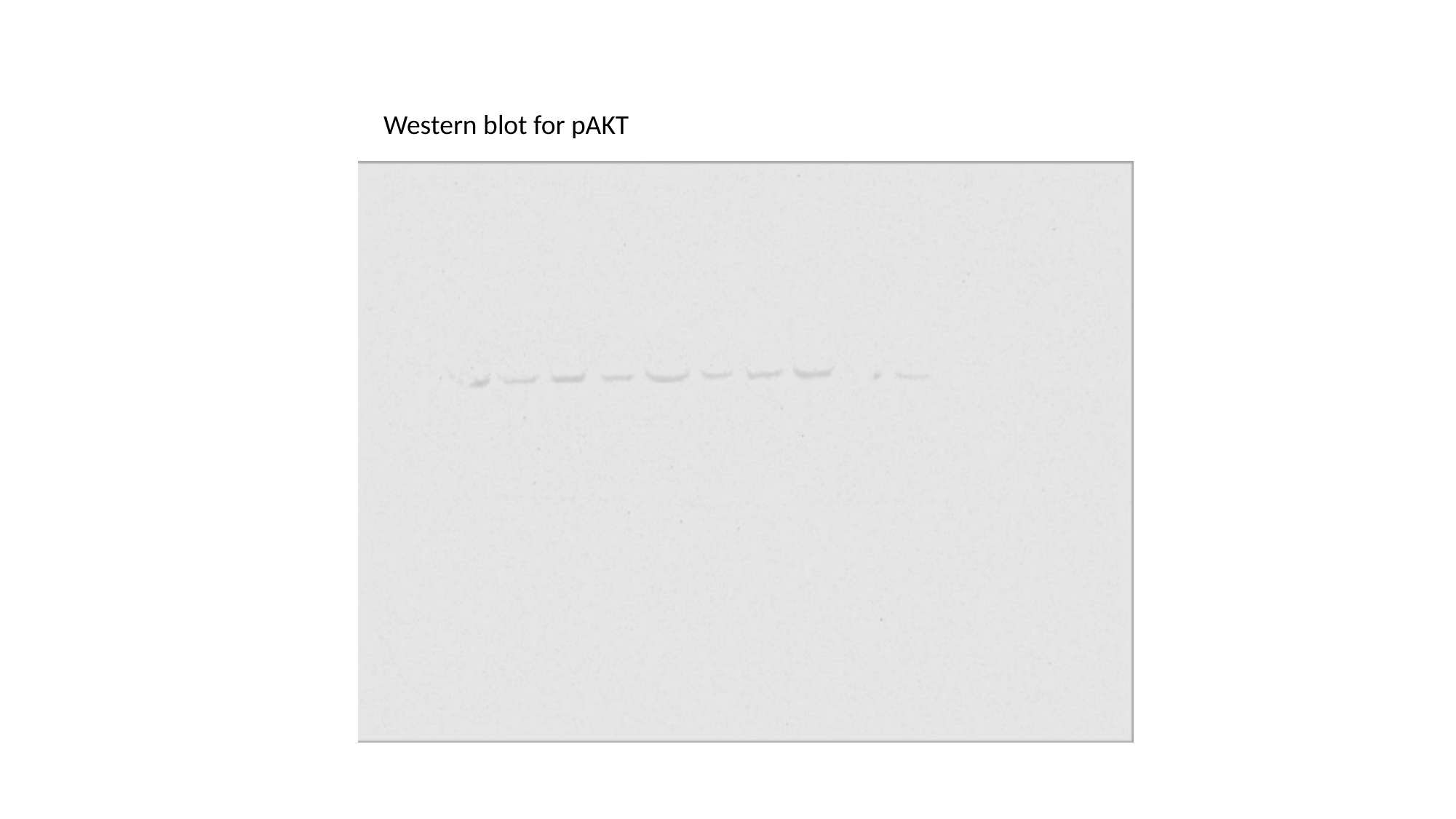

Western blot for pAKT

## Slide 2
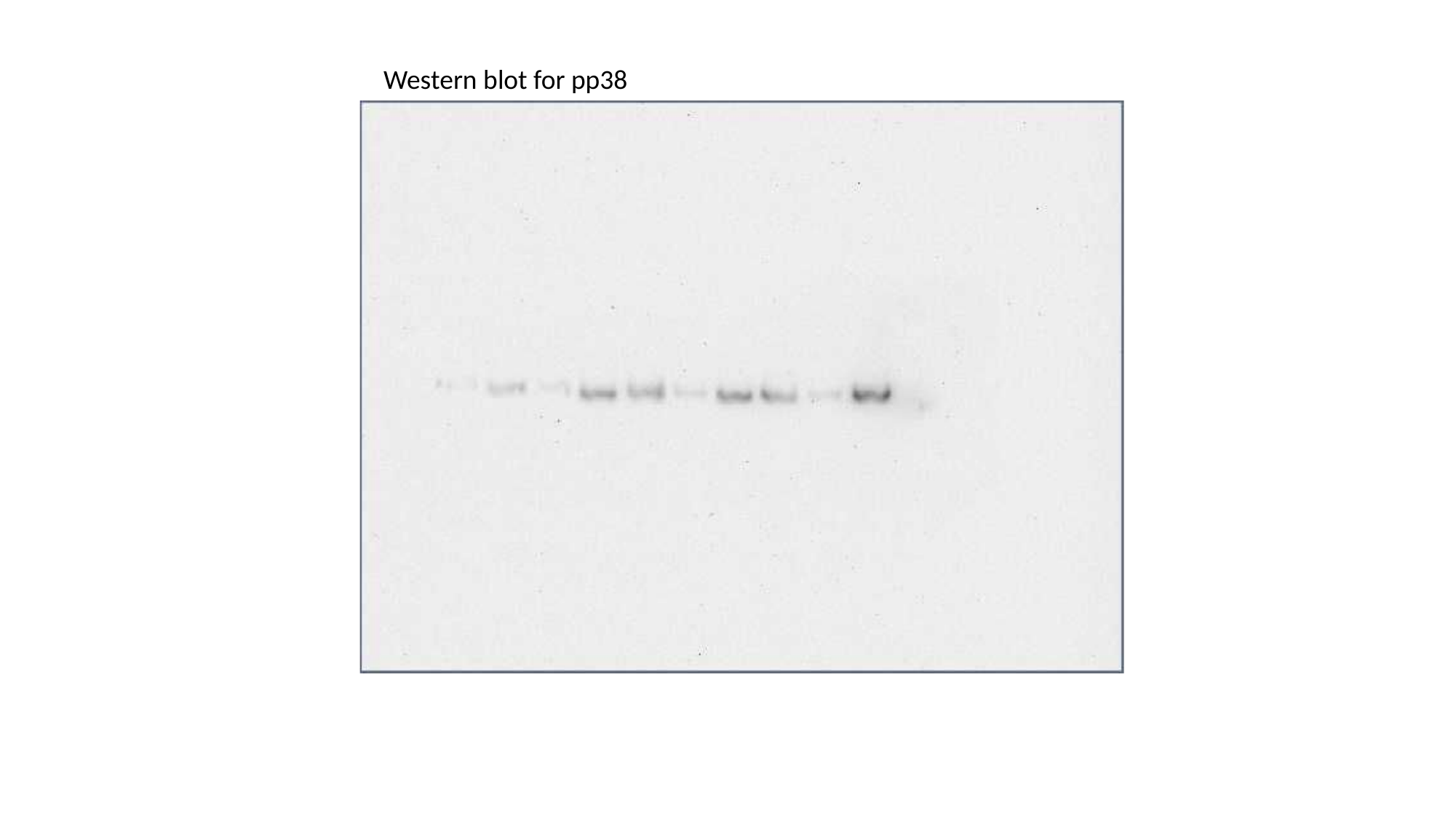

Western blot for pp38

## Slide 3
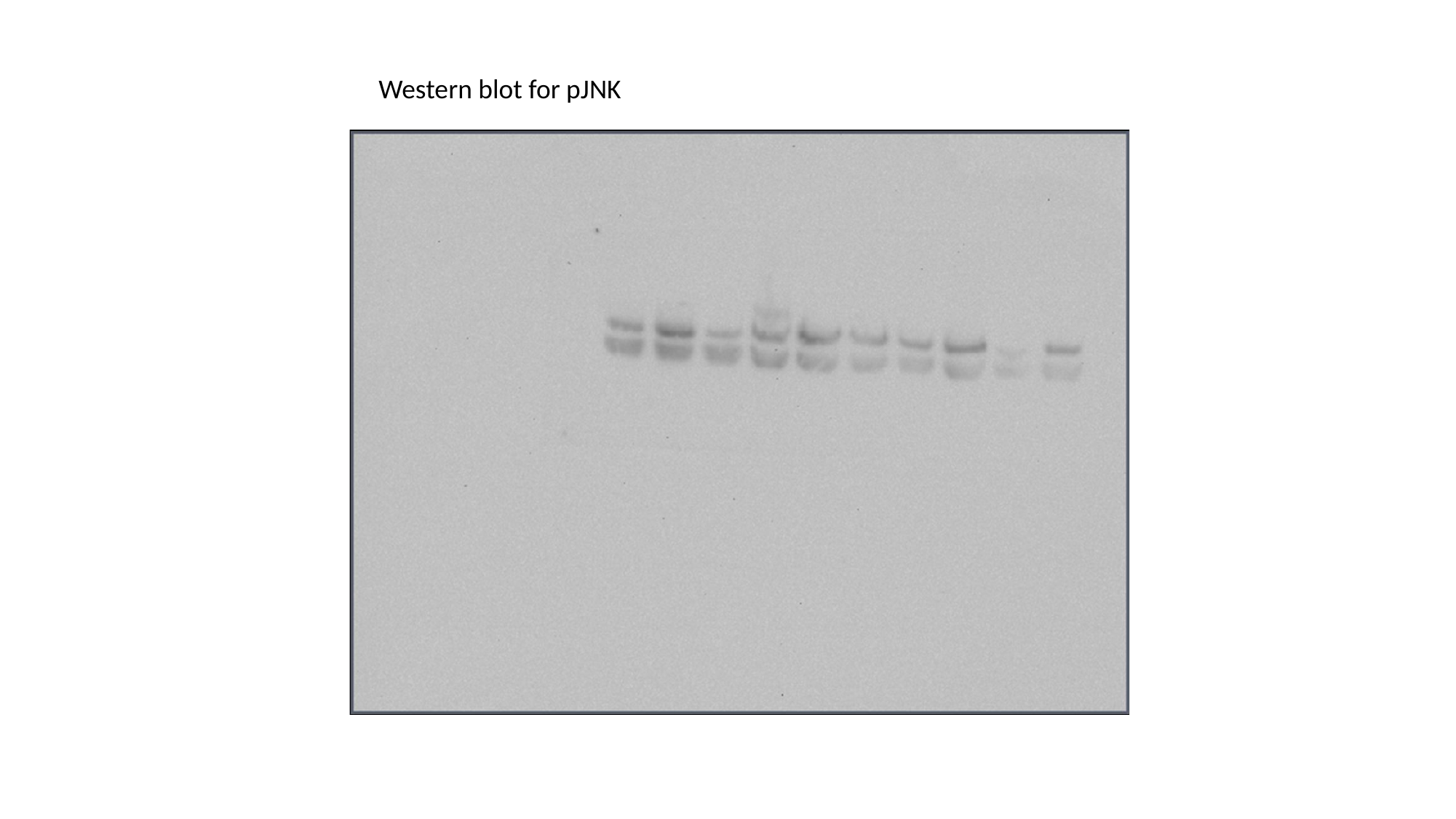

Western blot for pJNK

## Slide 4
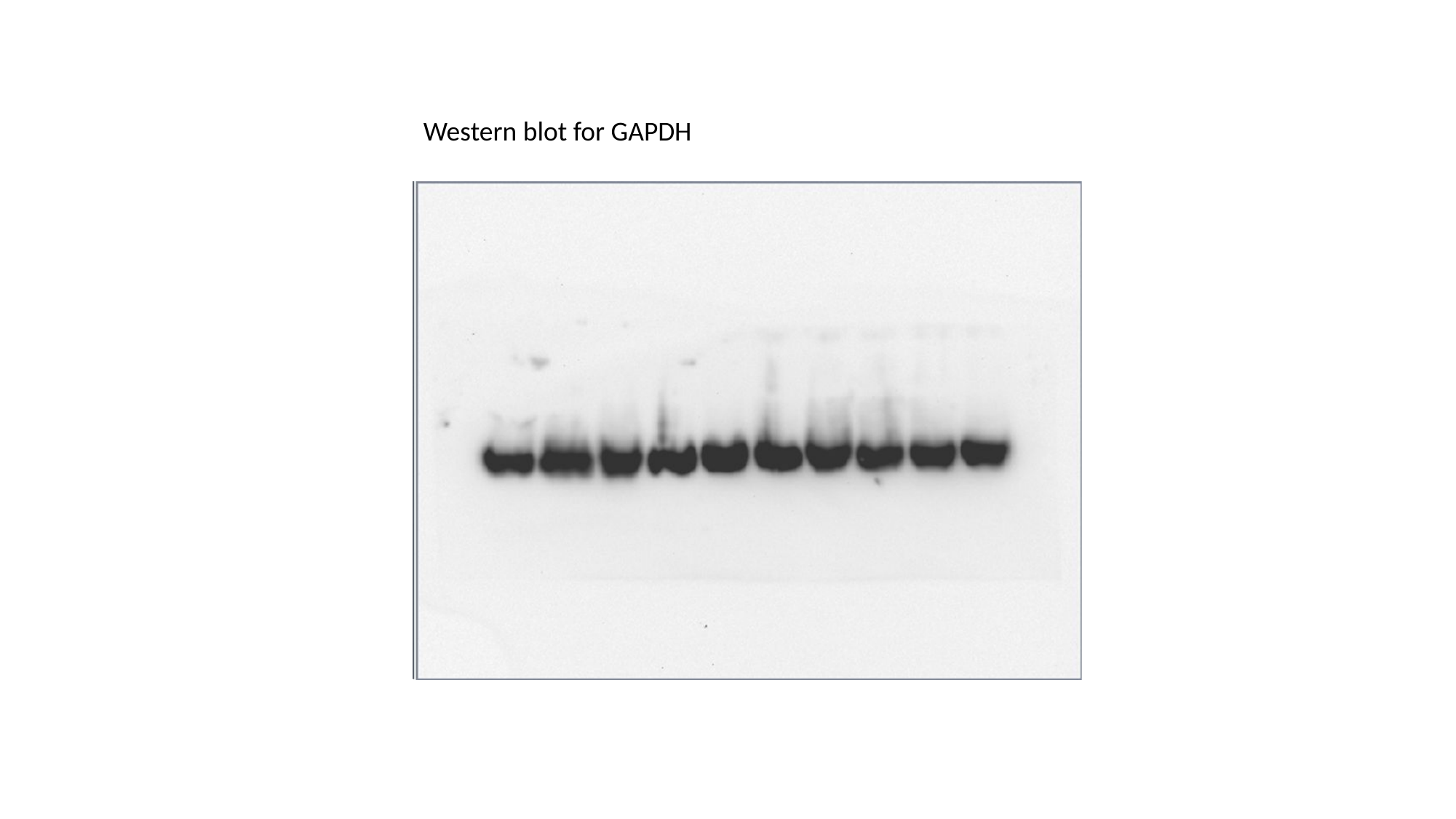

Western blot for GAPDH
